# Impact of perceived threats, benefits, and athlete brand image on attitudes toward naturalized athletes in the context of China

**DOI:** 10.3389/fpsyg.2024.1450073

**Published:** 2024-11-14

**Authors:** Haixiang Lu, Dongfeng Liu, Haozhou Pu, Alan Robert Persaud

**Affiliations:** ^1^School of Economics and Management, Shanghai University of Sport, Shanghai, China; ^2^School of Foreign Languages and Cultures, Southwest University of Science and Technology, Mianyang, China; ^3^Department of Health and Sport Sciences, University of Dayton, Dayton, OH, United States; ^4^School for Business and Society, University of York, York, United Kingdom

**Keywords:** naturalized athletes, athlete brand image, sport event involvement, likability, identification, support intention

## Abstract

**Introduction:**

China’s implementation of athletic naturalization has sparked controversy, yet empirical studies on the factors influencing public attitudes toward naturalized athletes remain scarce. This study aims to address this gap by constructing a theoretical model based on social identity theory, examining the impact of various psychological variables on respondents’ attitudes toward naturalized athletes in China’s national teams.

**Methods:**

Data were collected from 704 online respondents. Descriptive and exploratory factor analyses (EFA) were conducted using SPSS 25.0. Structural equation modeling with partial least squares (PLS-SEM) was employed to test the proposed hypotheses, utilizing SmartPLS 3.0.

**Results:**

The findings indicate that perceived threats negatively impact attitudes (*β* = −0.134, *p* < 0.001), while perceived benefits positively influence athlete brand image (*β* = 0.494, *p* < 0.001) and attitudes (*β* = 0.169, *p* < 0.001). Athlete brand image positively affects attitudes (*β* = 0.623, *p* < 0.01) and mediates the relationship between perceived benefits and attitudes. Additionally, sport event involvement positively moderates the relationship between athlete brand image and attitudes. The model explained 33.7% of the variance in athlete brand image and 68.5% of the variance in attitudes.

**Discussion:**

These findings suggest that naturalization decisions should consider the candidates’ overall brand image, with the naturalization process being more transparent and the naturalized athletes’ contribution being effectively communicated to the public. Additionally, fostering public involvement in sport events can create a more favorable atmosphere for athletic naturalization.

## 1 Introduction

Globalization has significantly influenced the development of modern sport, particularly since the 1990s, when the migration of athletes became more frequent due to the increasing professionalization and internationalization of sports leagues and events (Leng et al., 2021). Many countries have adopted the practice of recruiting foreign-born athletes to represent their national teams, a trend observed in major sporting events such as the FIFA World Cup and the Olympic Games (Maguire and Falcous, 2010; Maoski et al., 2021; Reiche and Tinaz, 2019). This has led to the widespread implementation of athletic naturalization strategies, including in China.

Despite China’s ambitious goals of becoming a powerful sporting country and using sports as a symbol of the Great Rejuvenation of the Chinese Nation (General Office of the State Council of the PRC, 2019), the structural weaknesses within its elite sports system have posed significant challenges. As the host of several global mega-events, China aims to deliver outstanding performances but faces difficulties achieving this through its existing athlete training and selection systems alone.

Against this backdrop, recruiting foreign sporting talents to represent China is viewed as an expedited solution. Naturalized athletes are those who voluntarily acquire the nationality of a country other than their birthplace, gaining membership in its sports associations and competing internationally under that nation’s flag (Lin, 2013). To date, China has particularly focused on recruiting naturalized athletes in preparation for events such as the FIFA World Cup and Winter Olympics (see Table 1).

**Table 1:** Naturalized athletes who represented China (2019–2024).

Name	Sport	Original nationality
Fei Nanduo (Fernando), Jiang Guangtai (Tyias Browning), and other 4 athletes	Men’s football	Brazil/UK
Ye Jinguang (Brandon Yip), Zheng Enlai (Ty Schultz), and other 13 athletes	Men’s ice hockey	Canada/US/Russia
Zhou Jiaying (Kimberly Newell), le MI (Hannah Miller), and other 11 athletes	Women’s ice hockey	Canada/US
Lin Xiaojun (Lim Hyojun), Liu Shaolin (Shaolin Sandor Liu), Liu Shaoang (Shaoang Sandor Liu)	Men’s short track Speed skating	South Korea/Hungary
Zhu Yi (Beverly Zhu), Lin Shan (Ashley Lin)	Women’s figure skating	US
Gu Ailing (Eileen Gu)	Women’s freestyle skiing	US
Zheng Ninali (Nina Schultz)	Women’s heptathlon	Canada
Li Kaier (Kyle Anderson)	Men’s basketball	US

These naturalized athletes are considered strategic assets for enhancing the sporting competitiveness of their adopted countries, often requiring significant investments. To maximize the value of these “assets,” it is crucial to secure broad public support, which can improve the athletes’ adaptability to a new society and enhance their performance (Kim et al., 2020). However, despite the growing presence of naturalized athletes in China’s national teams, this strategy has faced intense public scrutiny, with rising concerns about the athletes’ underlying motives and allegiance (Leng et al., 2021; Li et al., 2024).

In this context, athletic naturalization has become a prominent issue in academic discourse, prompting the need for further research to identify the factors and mechanisms shaping public perceptions of naturalized athletes. This study aims to construct a theoretical model based on social identity theory and to examine the impact of various psychological variables on individuals’ perceptions of naturalized athletes. The findings may contribute to the development of effective strategies for managing the naturalization of athletes in countries seeking to diversify their selection systems, enhance sporting competitiveness, and expand global influence. Moreover, this study could offer insights into a broader conceptualization of the “imagined community” (Anderson, 1983), as the perception of naturalized athletes is closely tied to the public’s multicultural acceptability (Lee, 2008; Lee et al., 2018).

## 2 Literature review and hypothesis development

### 2.1 Athletic naturalization in China and other countries

Athletic naturalization has been a contentious issue since its inception, particularly when athletes switch allegiances to represent nations with which they have minimal historical, ethnic, linguistic, or cultural ties (Grix, 2016).

In Japan for example, the failure of its ice hockey team to qualify for the Lillehammer Winter Olympics motivated the country to open its domestic sports system (Chiba et al., 2001). The Japanese Ice Hockey Federation (JIHF) began recruiting Canadian-born athletes of Japanese descent to improve the men’s national team’s performance at the 1998 Nagano Winter Olympics. Recruiting athletes of Japanese ancestry was a strategy employed by the JIHF to quickly build a stronger team and mitigate potential public backlash against fully foreign athletes without Japanese heritage. However, Japanese media criticized this naturalization as a shortsighted policy enacted at the expense of developing domestic players (Chiba et al., 2001).

Since 2013, South Korea has aggressively recruited foreign-born winter sports athletes, despite this contradicting its long-standing ethnic nationalism. The naturalization effort was part of South Korea’s nation-building project aimed at preparing competitive national teams for the 2018 PyeongChang Winter Olympics (Shin and Lee, 2021). To facilitate naturalization, the government relaxed the Nationality Law (Article 7) (Choi, 2020) and began allowing dual citizenship (Lee, 2010). As athletic naturalization progressed, the South Korean public became more accepting of white naturalized athletes than those of Black or other ethnic backgrounds, as the country’s racial hierarchy, which favors and normalizes white identity, reinforces racist views toward Black people and other people of color (Choi, 2020).

For Qatar, sports investment is a key part of its soft power strategy, centered around three themes: showcasing Qatar’s supremacy as a microstate; pursuing peace, security, and integrity; and addressing national health crises (Brannagan and Giulianotti, 2015). A major challenge to Qatar’s sporting success is its small population and low participation rates in sports, especially among women (Reiche, 2015). Naturalization is a key strategy to enhance Qatar’s visibility and success in international sports, and the number of naturalized athletes it has is significantly higher than international norms (Reiche and Tinaz, 2019). However, Qatar only grants these athletes temporary citizenship, unless they achieve exceptional success, suggesting that most foreign-born athletes are not seen as true members of Qatari society.

Like Qatar, Turkey is heavily investing in sports as a branding strategy to promote a positive image both domestically and internationally (Polo, 2015). Despite having a much larger population, Turkey faces challenges with low sports participation, a lack of facilities, and an underdeveloped school sports system (Tinaz et al., 2014). Since 2014, Turkey has expanded athletic naturalization after Turkish athletics collapsed due to widespread doping in 2013 (Reiche and Tinaz, 2019). Naturalizing foreign-born athletes has become a key strategy for Turkey in its quest to become a major sporting power.

While China has historically excelled in many Summer Olympic sports, the primary motivation for naturalizing foreign-born athletes is to strengthen its sporting prowess by addressing structural weaknesses in its elite sports system (Hai et al., 2021). This strategy also serves as a response to the threats from other countries, such as the naturalization of footballers in Indonesia and the Philippines, within the broader context of global athletic naturalization. Different from the aforementioned countries, China has achieved a leading position in the recent Summer Olympics and Asian Games (General Administration of Sport of China, 2021), recruiting foreign-born sporting talents is an efficient strategy to improve its overall sporting competitiveness and keep the dominant position in mega sport-events. In the context that China does not encourage immigration, accept refugees or asylum seekers (Sullivan et al., 2023), the major obstacles to the development of athletic naturalization for China include the citizens’ exclusive inclination based on the ethnic nationalism (Huang et al., 2021; Ni and Wang, 2020), and the limitation of the nationality law (doctrine of single nationality) (Liang et al., 2020; Qian, 2020; Xu et al., 2019). Scholars generally argue the direct benefits brought by naturalized athletes for the adopting country include improving its international competitiveness, intensifying the domestic competition and consequently improving the indigenous athletes (Hai et al., 2021; Lin, 2013; Ni and Wang, 2020). Academics have also expressed their concerns that the extensive employment of athletic naturalization may restrain the development of the youth training system (Chen and Wang, 2020; Lin, 2013), undermine the public’s national pride from elite sport (Lin, 2013; Zhang, 2019), and even blur national identity (Huang, 2014).

### 2.2 Attitudes toward the naturalized athletes

Given that people’s association with athletes can influence their team identification and involvement in certain sports (Funk et al., 2001; Hong et al., 2005; Wu et al., 2012), their perceptions of naturalized athletes may significantly affect their relationships with the athletes’ teams, leagues, sponsors, and even the elite sports system as a whole (Kunkel et al., 2020). Thus, understanding public perceptions of naturalized athletes is of critical importance.

To date, various constructs have been employed to measure public perceptions of naturalized athletes, including likability (Kim et al., 2015; Kim et al., 2020; Lee, 2008), positive/negative sentiment (Leng et al., 2021), acceptance (Willis et al., 2010), national identity and perceived similarity (Stelzl et al., 2008).

The previously mentioned constructs do not fully capture the public’s psychological perception of naturalized athletes. Therefore, this study employs the construct of attitude to assess respondents’ overall perception, encompassing affect, cognition, and behavioral intention (Mcdougall and Munro, 1987). Attitude is a hypothetical construct that reflects a general and enduring positive or negative feeling toward, or evaluative response to, a person, object, or issue (Petty and Cacioppo, 1986). The affective component of attitude refers to an individual’s feelings toward a specific object or scenario, which can range from pleasurable to unpleasurable (Schwarz, 2000). The cognitive component consists of beliefs, knowledge structures, perceptual responses, and thoughts, varying from favorable to unfavorable (Breckler, 1984). The behavioral component embodies overt actions, behavioral intentions, and verbal statements regarding behavior, which can range from favorable and supportive to unfavorable and hostile (Breckler, 1984). Therefore, attitude is proposed as the appropriate construct to capture the complete psychological perception of naturalized athletes. Specifically, likability, identification, and support intention are used to represent the three dimensions of attitude.

*Likability* measures respondents’ affection for the naturalized athlete, as interpersonal attraction and liking are central to the affective component of attitude (Liden et al., 1993). Erdogan (1999) defines likability as a source of affection based on physical appearance and behavior. In this study, likability refers to positive feelings and goodwill toward the athlete, reflecting affection for the athlete’s behavior or personality traits in the context of sports (Kim et al., 2020; Feltham et al., 1991).

*Identification* is used to measure respondents’ cognitive connection to the specified naturalized athlete, as it reflects the cognitive structures organized by their knowledge, beliefs, and expectations (Merick, 2005). Identification with an athlete involves individuals perceiving a personal link with the athlete, where the athlete’s achievements and setbacks are seen as personal experiences (Wu et al., 2012). This identification indicates a deep psychological attachment to the athlete. Stelzl et al. (2008) expanded on the self-management techniques of BIRGing (basking in reflected glory) and CORFing (cutting off reflected failure) by adopting a social identity perspective. These techniques, which typically focus on associating or dissociating oneself from another person or group, can also be applied to others rather than just the self. In this context, in-group members may include another individual within the in-group by emphasizing shared social identity when the individual achieves something positive or exclude them by highlighting their non-shared identity when they do something negative. Accordingly, in this study, identification with the naturalized athlete is operationalized as the willingness to include these athletes as members of the in-group (i.e., the Chinese nation) and to share in their successes.

*Support intention* measures respondents’ behavioral intentions toward naturalized athletes, as intention is a consistent predictor of future behaviors (Fishbein and Ajzen, 1975). Extant literature suggest the attachment to a favorite athlete can influence spectators’ behaviors (Mahony et al., 2002). Support intention is assessed by examining respondents’ sport consumption intentions and behavioral loyalty, including game attendance, viewership, media and merchandize consumption, participation in discussions, and word-of-mouth related to the athletes (Braunstein and Zhang, 2005; James and Trail, 2008; Mahmoudian et al., 2021; Shapiro et al., 2013; Shin and Lee, 2021).

### 2.3 Factors influencing perception of naturalized athletes

Scholars have explored the factors influencing public acceptance of ethnic minorities in national representative roles from various perspectives. From a macroscopic view, historical, economic, and political differences among nations shape the overall receptiveness of their populations to such minorities (Choi, 2020). For instance, East Asian researchers emphasize the role of ethnic composition, noting that ethnically homogeneous societies tend to be more exclusionary toward immigrants, including naturalized athletes (Chiba et al., 2001; Lee, 2008; Maeng and Cho, 2012). In these societies, the myth of pure blood, territorial affiliation, and monolingualism serve as rigid ideological components that construct an exclusive national identity (e.g., Chineseness, Koreanness, Englishness) (Choi, 2020). Conversely, in regions aiming to construct a more inclusive national identity (e.g., Hong Kongness), there is greater flexibility in public attitudes: concepts such as “internationalness” justify ethnic and racial diversity by recognizing citizenship and adoption of local customs as pathways to belonging (Chiu, 2021); the “instrumental-plus” concept measures the representativeness of naturalized athletes by their contributions plus additional factors, such as familiarity with local culture and identification with the region as home (Yung et al., 2021); the “civic-plus” orientation prioritizes rational-legal citizenship and equal treatment, valuing allegiance over ethnic and racial ties, thus allowing for a more inclusive acceptance of naturalized athletes (Yung et al., 2021).

Within a country’s unique historical and cultural context, certain sports are particularly emblematic arenas for the expression of nationalistic sentiments, where public opinion often disfavors the recruitment of naturalized athletes. Marathons in South Korea (Choi, 2020) and football in England (Gibbons, 2015) serve as key examples.

From the perspective of individuals in host countries, scholars argue that national identity is closely linked to elite sports at the national level (Burdsey, 2006; Fletcher, 2012; Maguire et al., 1999). Consequently, national identity may be a significant factor influencing attitudes toward naturalized athletes, aligning with immigration studies that suggest the populace’s national identity strongly impacts their views on immigrants (Bikmen, 2015; Louis et al., 2013). Furthermore, acceptance levels vary across different demographic groups (Cho and Lee, 2018; Willis et al., 2010).

Despite existing literature addressing factors from macro-level national perspectives to micro-level individual considerations, the psychological and perceptual dimensions of sports remain underexplored in explaining the gaps between national identity and public perceptions of naturalized athletes. As a result, practical solutions to enhance public acceptance are difficult to develop. To address these gaps, we introduce the constructs of perceived threats, perceived benefits, athlete brand image, and sport event involvement. These elements are incorporated into a theoretical model designed to elucidate the mechanism by which individuals form attitudes toward naturalized athletes.

### 2.4 Effect of perceived threats and perceived benefits on attitudes

This study is grounded in social identity theory, which provides a comprehensive framework for examining intergroup relations. Social identity is defined as “part of an individual’s self-concept, derived from their knowledge of membership in a social group, along with the value and emotional significance attached to that membership” (Tajfel, 1981, p. 255). Social identity theory posits that individuals seek to enhance or maintain their self-concept or self-esteem by attributing positive qualities to their in-group and negative qualities to out-groups (Tajfel and Turner, 1979). This theory has been widely applied in research on public perceptions of immigrants (Esses et al., 2006; Kessler et al., 2010) and foreign-born athletes (Stelzl et al., 2008). In line with this framework, we introduce the constructs of perceived threats and perceived benefits in this study to investigate the specific causes leading to positive or negative attitudes toward naturalized athletes.

The concept of *perceived threats* is rooted in the integrated threat theory of prejudice (Stephan et al., 2000), which is frequently employed to study intergroup relations in culturally plural societies, such as local citizens’ attitudes toward immigrants. Perceived threats can be categorized as either realistic or symbolic. Realistic threats involve perceptions that the out-group endangers the in-group’s existence, political or economic power, or physical wellbeing; while symbolic threats pertain to the belief that the out-group jeopardizes the in-group’s “way of life,” including values, customs, traditions, beliefs, and attitudes (Bizman and Yinon, 2001). These threats can be experienced at various levels, with individuals feeling personally threatened or perceiving a threat to their in-group as a whole (McLaren, 2003). As perceived threats increase, members of the receiving society often exhibit more negative attitudes toward immigrants (Louis et al., 2013; Stephan et al., 2000; Ward and Masgoret, 2006). Similarly, citizens of an adopting country may perceive athletic naturalization as a threat, expressing concerns such as “depriving local players of opportunities to become national players” or “causing the national identity crisis” (Cho and Lee, 2018, p. 461).

In contrast, *perceived benefits* refer to beliefs about the positive outcomes associated with a behavior, particularly in response to a real or perceived threat (Tingchi Liu et al., 2013). While individuals may perceive benefits from immigrants (Maeng and Kwon, 2015; Pereira et al., 2010), this construct has not been extensively tested in existing immigration studies. Athletic naturalization represents a unique phenomenon distinct from general immigration, often driven by strategic objectives set by sports administrators. The positive and negative effects of athletic naturalization are likely to be directly perceived by individuals based on their sports identity, which is associated with specific sports, teams, or athletes (Funk and James, 2004). For example, the belief that naturalized footballers will enhance the national team’s performance is correlated with positive attitudes toward these athletes (Willis et al., 2010).

Given that naturalized athletes can be classified as immigrants, we hypothesize that perceived threats associated with their introduction will lead to negative attitudes, while perceived benefits will foster positive attitudes. Accordingly, the following hypotheses are proposed:

*H1*: Perceived threats have a negative impact on attitudes toward naturalized athletes.

*H2*: Perceived benefits have a positive impact on attitudes toward naturalized athletes.

### 2.5 Effect of athlete brand image on attitudes

Although numerous studies have examined factors influencing public perceptions of naturalized athletes, research specifically addressing the impact of athletes’ attributes—particularly their brand image—on respondents remains limited. An athlete’s brand image refers to the set of associations that people identify with a particular athlete (Kunkel et al., 2020). Arai et al. (2013) developed the athlete brand image (ABI) model, which encompasses three major dimensions—*athletic performance*, *attractive appearance*, and *marketable lifestyle*—and 10 sub-dimensions (*athletic expertise*, *competition style*, *sportsmanship*, *rivalry*, *physical attractiveness*, *symbol*, *body fitness*, *life story*, *role model*, *relationship effort*).

This model provides one of the first comprehensive frameworks for understanding athletes’ brand image and suggests mechanisms to enhance ABI. Liu et al. (2016) adapted this model to the Chinese context, identifying six major dimensions: *external image*, *moral style*, *sports level*, *competition style*, *social responsibility*, and *relationship with fans*. The perception of an athlete is influenced by their overall brand image, which is constructed through both pre-existing and new information encountered by consumers, including fans (Arai et al., 2014). Consequently, perceptions of ABI may vary even for the same athlete.

The ABI has been employed to predict respondents’ psychological commitment, behavioral loyalty to specific athletes, and behavioral intentions toward products endorsed by these athletes (Mahmoudian et al., 2021; Shin and Lee, 2021; Väätäinen and Dickenson, 2019). Attitude is also frequently modeled as a dependent variable of image management (Bruner and Hensel, 1996; Homer, 2006; Kirmani and Shiv, 1998). Lee (2008) developed a scale for athlete image—comprising dimensions of sportiness, appearance, performance, and morality—in the South Korean context, demonstrating that sports fans’ perceptions of foreign athletes’ image (who are playing in South Korea) positively correlate with their likability. Kim et al. (2015) extended Lee’s work by confirming the causal relationship among perceptions of foreign athletes’ image, likability, and social distance. Additionally, Stelzl et al. (2008), through both archival research and experimental design, confirmed that an immigrant athlete’s performance achievements and reputation significantly influence media and public perceptions of the athlete’s national identity, determining whether they are recognized as a *bona fide* member of a nation.

Since the aforementioned factors are referred to the athletic expertise and sportsmanship associations of ABI, we integrate the ABI model with attitudes to test whether ABI similarly affects public attitudes toward naturalized athletes. Accordingly, the following hypothesis is proposed:

*H3*: ABI has a positive influence on attitudes.

### 2.6 Effect of perceived threats and perceived benefits on athlete brand image, and the mediating effect of athlete brand image

Perceived threats are considered to foster prejudice against those perceived as threatening (Bizman and Yinon, 2001; Stephan et al., 2000). According to social identity theory (Tajfel and Turner, 1979), the social categorization of individuals into out-groups and an in-group triggers a motivation to maintain or achieve positive group distinctiveness. This often involves enhancing the image, prestige, and resources of one’s own group by derogating or discriminating against out-groups (Esses et al., 2006). For instance, individuals with traditional racist views may perceive ethnic minorities as generally threatening and, as a result, disparage them in various contexts (Kessler et al., 2010).

Given that image is a direct and perceptible attribute of naturalized athletes, we hypothesize that when individuals feel threatened by athletic naturalization, they are likely to derogate the athletes’ image. Conversely, perceived benefits may motivate individuals to hold a positive perception of these athletes’ brand image, facilitating their inclusion as in-group members and thereby enhancing the in-group’s status. Accordingly, we propose the following hypotheses:

*H4*: Perceived threats have a negative influence on the naturalized athlete’s brand image.

*H5*: Perceived benefits have a positive influence on the naturalized athlete’s brand image.

Feeling threatened by a group may not automatically translate into a desire to completely exclude immigrants from society (McLaren, 2003), suggesting that mediators may exist between perceived threats/benefits and attitudes. As Lee et al. (2018) indicated, the image of naturalized athletes mediates perceptions of changing citizenship and multicultural acceptability. Therefore, we propose that ABI serves as a mediator between perceived threats/benefits and attitudes, leading to the following hypotheses:

*H6*: ABI mediates the relationship between perceived threats and attitudes.

*H7*: ABI mediates the relationship between perceived benefits and attitudes.

### 2.7 Moderating effect of sport event involvement

Involvement is defined as “a person’s perceived relevance of the object based on inherent needs, values, and interests” (Zaichkowsky, 1985, p. 342). Specifically, Trivedi (2020, p. 193) describes sport involvement as “an unobservable state of motivation, arousal, or interest in viewing a game or participating in a sport-related activity that results in searching, information-processing, and decision-making.” Sport involvement can be measured by enduring involvement (EI) that reflects an individual’s ongoing interest in sport in general or a specific sport, or situational involvement (SI) which might be invoked by a game or event (Koo and Lee, 2019).

Involvement is recognized as a significant predictor of attitude and behavior, individuals with high involvement are more likely to proactively seek and process information about an object, leading to more favorable attitudes and behaviors (Zhang et al., 2021). Sport involvement can drive desired consumption behaviors, such as game attendance and viewership (Funk et al., 2004; Shank and Beasley, 1998), and serves as an antecedent to psychological commitment and behavioral loyalty (Bee and Havitz, 2010; Doyle et al., 2013). Its moderating effects have been documented in existing literature (Koo and Lee, 2019; Lardinoit and Derbaix, 2001). For instance, Trivedi (2020) confirmed that sport involvement moderates the relationship between corporate image and brand love.

Regarding the impact of sport involvement on attitudes toward naturalized athletes, Chiu (2021) argued that football fans (who are more involved) typically have greater prior knowledge of naturalized footballers’ Hong Kongness, leading to a greater willingness to accept these athletes as members and representatives of Hong Kong compared to non-football fans (who are less involved). Willis et al. (2010) found that respondents’ interest in football (i.e., the attraction dimension of involvement) is positively associated with their acceptability of naturalized footballers.

Given that events like the Olympics serve as significant platforms for nations to elevate their international recognition and promote patriotic nationalism (Lee and Maguire, 2009), and considering China’s proactive recruitment of foreign-born athletes in preparation for the Beijing Winter Olympics, we include sport event involvement (SEI)—reflecting respondents’ sense of personal relevance to and interest in a particular event (Wong and Tang, 2016)—in this study, guided by the contact hypothesis. The contact hypothesis (Pettigrew and Tropp, 2000) posits a positive relationship between intergroup contact and favorable social and psychological outcomes: Increased contact with minority group immigrants is associated with more inclusive attitudes toward immigration (McLaren, 2003; Stephan et al., 2000; Ward and Masgoret, 2006). Accordingly, we hypothesize that individuals highly involved with the Beijing Winter Olympics are likely to have more prior knowledge of athletic naturalization (Maeng and Kwon, 2014) and, through exposure to games, news media, and advertisements, will have increased “contact” with naturalized athletes representing China. This contact may result in more positive perceptions of these athletes’ brand image and more inclusive attitudes. Therefore, we propose the following hypotheses to examine the moderating effect of sport event involvement:

*H8*: Sport event involvement moderates the relationship between perceived threats and attitudes.

*H9*: Sport event involvement moderates the relationship between perceived benefits and attitudes.

*H10*: Sport event involvement moderates the relationship between ABI and attitudes.

**Figure 1:**
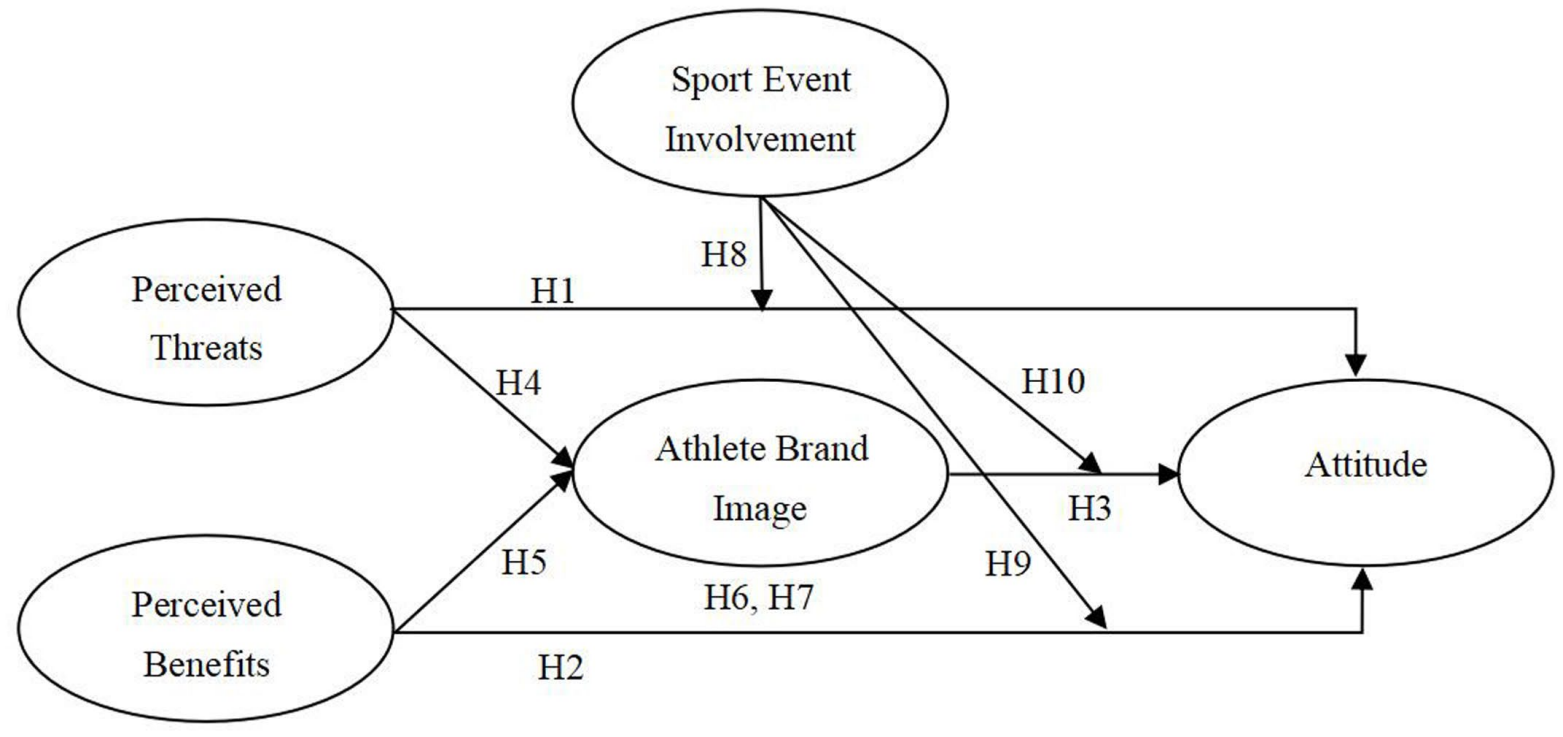
The hypothesized model.

The hypothesized model is presented in Figure 1.

## 3 Methods

### 3.1 Measures

A questionnaire was developed for online data collection, with all items designed to measure the constructs in this study adapted from previous research and modified to fit the context of naturalized athletes and the Winter Olympics. The constructs included in the ABI model were athletic expertise (AE), role model (RM), sportsmanship (SS), external image (EI), competition style (CS), and relationship effort (RE). The constructs of rivalry and life story were excluded, considering their limited relevance to the unique identity of naturalized athletes and the respondents’ likely familiarity with them. Following Kunkel et al. (2020), ABI was treated as a reflective-formative hierarchical component, as the lower-order components are not conceptually interchangeable and do not necessarily covary.

The questionnaire comprised 12 first-order constructs and two second-order constructs (ABI and attitude). Specifically, sport event involvement (SEI) was measured using 6 items adapted from Zhang et al. (2021); perceived threats were assessed using 5 items adapted from Cho and Lee (2018), Maeng and Cho (2012), and Maeng and Kwon (2014); perceived benefits were measured using 5 items adapted from Maeng and Kwon (2015) and Pereira et al. (2010). The 21 items related to ABI were sourced from Arai et al. (2013), Liu et al. (2016), and Kim et al. (2015). The attitude construct was divided into three dimensions: likability (measured with 3 items adapted from Kim et al., 2020), identification (5 items modified from Heere and James, 2007, and Stelzl et al., 2008), and support intention (5 items adapted from Bauer et al., 2008; Braunstein and Zhang, 2005; Shin and Lee, 2021). All constructs were measured on a five-point Likert scale ranging from 1 (“strongly disagree”) to 5 (“strongly agree”). Additionally, the questionnaire included items on respondents’ demographics and their awareness of naturalized athletes. Given that some items were sourced from English and Korean literature, they were translated into Chinese following Brislin’s (1980) back-translation procedure to ensure linguistic accuracy.

The content and face validity of the items were evaluated by a panel of experts. Five sport management academics were provided with detailed information about the research objectives, along with a list of constructs, their definitions, and associated items. The academics rated each item on a five-point Likert scale ranging from 1 (“does not reflect the construct at all”) to 5 (“reflects the construct very well”). As a result, 4 items were deleted due to receiving an average rating below 4 (Polit and Beck, 2006), leaving a total of 47 items.

### 3.2 Sample and data collection

The online survey was conducted on a data collection platform^1^ from January 27th (1 week before the 2022 Beijing Winter Olympics) to March 20th, 2022 (1 month after the event), to account for potential changes in respondents’ awareness as the event progressed. Participants were recruited online, targeting individuals who either intended to watch or had already watched the Beijing Winter Olympics. A list of eight naturalized athletes expected to compete in the Beijing Winter Olympics was provided (Gu Eileen, Zhu Yi, Lin Shan, Lin Xiaojun, Zheng Enlai, Ye Jinguang, Zhou Jiaying, Wang Yuting). Participants were first asked to select the athlete they were most familiar with from the list. Those who selected “I have no knowledge of any winter sports naturalized athlete” were excluded from further analysis.

A total of 1,106 questionnaires were collected. After careful examination, 402 questionnaires were eliminated due to inconsistent responses, highly repetitive answers, or unrealistically short response times. This resulted in 704 usable responses (usable rate 63.65%). The demographic profile of the respondents is presented in Table 2. The freestyle skiing athlete Eileen Gu was identified as the most familiar naturalized athlete by the majority of respondents (65.7%), followed by Lin Xiaojun (13.1%), Zhu Yi (10.9%), Lin Shan (6.0%), Zheng Enlai (3.3%), and Wang Yuting (1.0%).

**Table 2:** Demographics of respondents (*n* = 704).

Demographics	Frequency	(%)
Gender		
Male	293	41.6
Female	411	58.4
Age range		
<18	5	0.7
18–24	406	57.6
25–30	179	25.4
31–40	80	11.3
41–50	27	3.8
≥51	7	0.9
Educational level		
Junior high school and below	9	1.2
High school/Technical secondary school	16	2.2
Junior college	30	4.2
Undergraduate	493	70
Postgraduate and above	156	22.1
Income (CNY/Month)		
≤2,999	421	59.8
3,000–3,999	26	3.7
4,000–6,999	99	14.1
7,000–9,999	58	8.2
10,000–14,999	62	8.8
15,000–19,999	15	2.1
≥20,000	23	3.3

### 3.3 Data analysis

SPSS 25.0 was utilized to perform descriptive analysis and exploratory factor analysis (EFA) on the sample data. To test the proposed hypotheses, structural equation modeling with partial least squares (PLS-SEM) was employed using SmartPLS 3.0. PLS-SEM was considered an appropriate technique for this study for several reasons. First, given that respondents were recruited online, the sample is assumed to be non-normal, and PLS-SEM is well-suited for handling data that do not adhere to strict normal distribution requirements. Second, PLS-SEM allows researchers to model multiple interrelated dependence relationships within a single framework. Additionally, it accommodates both formative and reflective measures (Hair et al., 2011), making it ideal for the complex constructs examined in this study.

## 4 Results

### 4.1 Measurement model

The measurement models were first examined to verify the psychometric properties of the constructs used in this study (see Table 3). EFA was conducted to assess the validity of the items for each construct. As a result, one item from AE and one item from SI were deleted due to poor factor loading. Additionally, two items from identification and another item from SI were removed due to cross-loading on multiple factors.

**Table 3:** Psychometric properties of measurement models.

Latent construct and items	*M*	*SD*	Factor loading
1. *Sport event involvement* (AVE = 0.796, CR = 0.951, Cronbach’s α = 0.936)			
I paid (would like to pay) attention to the Beijing Winter Olympics.	3.54	1.02	0.865
Watching the Beijing Winter Olympics is important to me.	3.21	1.20	0.882
It is important for me to be involved with the Beijing Winter Olympics.	3.20	1.16	0.920
Spending time on the Beijing Winter Olympics is worthy.	3.59	1.14	0.881
It is my interest to watch the Beijing Winter Olympics.	3.22	1.15	0.911
2. *Perceived threats* (AVE = 0.837, CR = 0.953, Cronbach’s α = 0.935)			
Naturalizing foreign-born athletes would hinder the training and development of Chinese-born athletes.	2.40	1.18	0.901
Naturalizing foreign-born athletes is negative for the fairness of sports.	2.20	1.18	0.913
Naturalizing foreign-born athletes would depreciate people’s national pride from the national teams.	2.28	1.25	0.930
Naturalizing foreign-born athletes would cause the national identity crisis.	2.23	1.20	0.915
3. *Perceived benefits* (AVE = 0.864, CR = 0.962, Cronbach’s α = 0.948)			
Naturalizing foreign-born athletes would facilitate the development of domestic elite sports.	4.25	0.95	0.925
Naturalizing foreign-born athletes would make the domestic elite sports more vibrant.	4.26	0.95	0.950
Naturalizing foreign-born athletes would improve China’s competitiveness in international sport events.	4.28	0.93	0.916
Naturalizing foreign-born athletes would enrich China’s sport culture by bringing in new ideas and culture.	4.32	0.90	0.927
4. *Athlete brand image*			
*Athletic expertise* (AVE = 0.848, CR = 0.957, Cronbach’s α = 0.939)			
The athlete has prominent athletic skills in his/her sport.	4.67	0.67	0.945
The athlete is a dominating player in his/her sport.	4.62	0.73	0.951
The athlete is accomplished in his/her sport.	4.67	0.71	0.940
The athlete contributes a lot to the national team.	4.50	0.85	0.843
*Role model* (AVE = 0.867, CR = 0.963, Cronbach’s α = 0.948)			
The athlete has no scandals.	4.39	0.87	0.905
The athlete is socially responsible.	4.34	0.88	0.930
The athlete is good role model for others.	4.48	0.81	0.932
The athlete demonstrates good moral standards in our society.	4.43	0.82	0.956
*Sportsmanship* (AVE = 0.921, CR = 0.979, Cronbach’s α = 0.971)			
The athlete strives to win in competition.	4.62	0.71	0.959
The athlete shows fair play.	4.62	0.70	0.967
The athlete shows sportsmanship.	4.63	0.68	0.967
The athlete shows respect for his/her opponents and other players.	4.61	0.69	0.946
*External image* (AVE = 0.875, CR = 0.965, Cronbach’s α = 0.952)			
The athlete is physically attractive.	4.42	0.84	0.942
The athlete is good-looking.	4.34	0.85	0.940
The athlete is stylish.	4.40	0.83	0.945
The athlete is in good shape.	4.49	0.77	0.913
*Competition style* (AVE = 0.881, CR = 0.957, Cronbach’s α = 0.932)			
The athlete’s competition style is distinctive from other players.	4.10	0.94	0.903
The athlete’s competition style is charismatic.	4.31	0.88	0.960
The athlete’s competition style is exciting to watch.	4.30	0.91	0.952
*Relationship effort* (AVE = 0.880, CR = 0.956, Cronbach’s α = 0.931)			
The athlete shows appreciation for fans and spectators.	4.01	0.96	0.902
The athlete appears approachable to his/her fans.	4.27	0.86	0.957
The athlete tries to interact with fans.	4.25	0.88	0.954
5. *Attitude*			
*Likability* (AVE = 0.888, CR = 0.960, Cronbach’s α = 0.937)			
It is pleasurable to watch the athlete playing for China.	4.40	0.87	0.948
I am favorable to see the athlete becoming a member of the national squad.	4.42	0.85	0.962
I would like to make friends with the athlete.	4.38	0.91	0.916
*Identification* (AVE = 0.781, CR = 0.915, Cronbach’s α = 0.860)			
Despite the athlete represents China, I do not think he/she belongs to China.	3.93	1.12	0.868
The athlete is a real Chinese.	4.11	1.06	0.913
When talking about the athlete with people from other countries, I would like to use “We” to describe my relationship with the athlete.	4.17	1.05	0.869
*Support intention* (AVE = 0.881, CR = 0.887, Cronbach’s α = 0.933)			
I would like to follow the media reports about the athlete.	4.11	1.00	0.932
I would like to follow the athlete on social media.	3.85	1.17	0.943
I would like to participate in discussions of the athlete.	3.92	1.12	0.942

Following these adjustments, the factor loadings of the remaining 42 items all exceeded 0.60, indicating acceptable convergent validity (Hair et al., 1998). The Cronbach’s alpha and composite reliability (*CR*) values for all constructs exceeded the recommended threshold of 0.70 (Nunnally and Berstein, 1994), suggesting good reliability. The average variance extracted (*AVE*) for all first-order constructs exceeded the 0.50 cutoff, further supporting convergent validity (Bagozzi and Yi, 1988).

Additionally, the square roots of the *AVE* for each construct were greater than the corresponding intercorrelations between constructs (see Table 4), providing evidence of discriminant validity (Fornell and Larcker, 1981). Furthermore, all heterotrait-monotrait (HTMT) ratio correlations were below 0.90, meeting the discriminant validity criterion (Henseler et al., 2015).

**Table 4:** Square root of *AVE* (diagonal) and correlation matrix.

	SEI	PT	PB	AE	RM	SS	EI	CS	RE	LIK	IDE	SI
SEI	**0.892**											
PT	−0.102	**0.915**										
PB	0.303	−0.318	**0.930**									
AE	0.224	−0.191	0.445	**0.921**								
RM	0.265	−0.226	0.526	0.688	**0.931**							
SS	0.251	−0.215	0.499	0.653	0.773	**0.960**						
EI	0.229	−0.196	0.455	0.595	0.704	0.668	**0.935**					
CS	0.261	−0.223	0.518	0.677	0.802	0.761	0.693	**0.939**				
RE	0.251	−0.215	0.499	0.652	0.772	0.733	0.668	0.760	**0.938**			
LIK	0.347	−0.340	0.612	0.642	0.759	0.721	0.657	0.748	0.720	**0.942**		
IDE	0.271	−0.270	0.487	0.511	0.605	0.574	0.523	0.595	0.573	0.682	**0.884**	
SI	0.285	−0.273	0.491	0.516	0.610	0.579	0.528	0.601	0.579	0.688	0.548	**0.939**

### 4.2 Test of common method bias and multicollinearity

Given that all constructs were measured using the same questionnaire, common method bias (CMB) was a potential concern. To mitigate CMB, respondents were assured of confidentiality and anonymity, and a reverse item was included in the survey. The one-factor solution approach (Podsakoff et al., 2003) was employed to assess the presence of CMB. The results indicated a significantly lower model fit for the one-factor model (*χ*^2^/*df* = 20.390, CFI = 0.510, TLI = 0.486, NFI = 0.498, RMSEA = 0.166) compared to the original model (*χ*^2^/*df* = 2.759, CFI = 0.956, TLI = 0.953, NFI = 0.934, RMSEA = 0.050), suggesting that CMB was not a serious issue in this study.

Additionally, to check for potential multicollinearity issues, variance inflation factors (VIF) were examined. The VIF values were all below the recommended threshold of 5, indicating that multicollinearity was not a concern (Hair et al., 2011).

### 4.3 Structural model

A bootstrapping analysis with 5,000 resamples was conducted to assess the validity of the structural model and to determine the path significance of the hypotheses. Key metrics including path coefficients, coefficient of determination (*R*^2^), cross-validated redundancy (*Q*^2^), and effect size (*f*^2^) were used to evaluate the predictive ability of the hypothesized model.

The path coefficients and explained variance for the structural model are presented in Table 5. According to the results, Hypothesis 1 is supported: Perceived threats have a negative effect on attitudes (*β* = −0.134, *p* < 0.001). Hypothesis 2 is supported: Perceived benefits have a positive effect on attitudes (*β* = 0.169, *p* < 0.001). Hypothesis 3 is supported: ABI is positively correlated with attitudes (*β* = 0.623, *p* < 0.01). Hypothesis 4 is rejected: Perceived threats does not have a significant impact on ABI (*p* > 0.05). Hypothesis 5 is supported: Perceived benefits have a positive impact on ABI (*β* = 0.494, *p* < 0.001). The model explained 33.7% of the variance in ABI and 68.5% of the variance in attitudes, indicating moderate predictive power of the model.

**Table 5:** Path coefficients and explained variance of the structural model.

Hypotheses	Standardized path coefficient	*t*-value	LLCI	ULCI	Supported Yes/No
*H1* PT → ATT	−0.134***	4.892	−0.187	−0.078	Yes
*H2* PB → ATT	0.169***	4.824	0.098	0.236	Yes
*H3* ABI → ATT	0.623***	20.436	0.566	0.686	Yes
*H4* PT → ABI	−0.060	1.718	−0.133	0.004	No
*H5* PB → ABI	0.493***	11.919	0.414	0.578	Yes
Explained variance					
ABI		*R*^2^ = 0.337			
Attitudes		*R*^2^ = 0.685			

*p < 0.05; **p < 0.01; ***p < 0.001.

A blindfolding procedure with an omission distance of 7 was used to calculate *Q*^2^ values, and these results, along with *f^2^* values, are presented in Table 6. The *Q*^2^ value for each endogenous variable significantly exceeded the threshold value of 0, confirming the model’s predictive relevance and the significance of the structural relationships. In terms of effect sizes (*f*^2^), Cohen (1988) categorizes them as small (0.02), medium (0.15), and large (0.35). The results indicated the *f*^2^ effect size for the predictive value of perceived benefits on ABI was medium; the *f*^2^ of perceived threats and perceived benefits on attitudes were small; the *f*^2^ of ABI on attitudes was large. These findings confirm the robustness and significance of the structural model in predicting attitudes toward naturalized athletes.

**Table 6:** *Q*^2^ and *f*^2^ values of the latent variables.

	SSO	SSE	*Q* ^2^	*f* ^2^
ABI	15488.000	12269.180	0.208	PT → ABI: 0.005
				PB → ABI: 0.295
Attitudes	6336.000	3595.380	0.433	PT → ATT: 0.049
				PB → ATT: 0.056
				ABI → ATT: 0.815

### 4.4 Mediating effects analysis

The mediating effects of ABI were tested using a bootstrapping procedure. The results for indirect effects, total effects, and variance accounted for (VAF) are presented in Table 7. These findings show that Hypothesis 7 was supported: ABI partially mediated the relationship between perceived benefits and attitudes. However, Hypothesis 6 was rejected, as ABI did not mediate the relationship between perceived threats and attitudes.

**Table 7:** Indirect effect, total effect, and VAF.

Path	Indirect effect	Total effect	VAF	Supported?
H6 PT → ABI → ATT	−0.037	−0.171***	N/A	No mediation
H7 PB → ABI → ATT	0.307***	0.477***	64.36%	Partial mediation

VAF > 80% = full mediation, 20% < VAF < 80% = partial mediation, VAF < 20% = no mediation.

***p < 0.001.

### 4.5 Moderation effects analysis

To examine the moderating effects of SEI, a two-step approach was employed. First, the main effects of the model were assessed without the interaction term. In the second step, the interaction term was included to evaluate its effect on the dependent variables. The results indicated that SEI moderated the relationship between ABI and attitudes, supporting Hypothesis 10. However, Hypotheses 8 and 9 were rejected, as SEI did not moderate the relationships between perceived threats or perceived benefits and attitudes (see Table 8).

**Table 8:** Path coefficients of moderating variables and significant level.

Path	Path coefficient	*t*-value	CI	Supported?
H8 PT × SEI → ATT	0.049	1.516	[−0.014, 0.113]	No
H9 PB × SEI → ATT	−0.057	1.797	[−0.121, 0.004]	No
H10 ABI × SEI → ATT	0.106**	3.340	[0.045, 0.170]	Yes

**p < 0.01.

## 5 Discussion

### 5.1 Theoretical contribution

The present study aims to contribute to the growing body of knowledge on athletic naturalization by developing strategies to enhance public attitudes toward naturalized athletes from a psychological perspective.

Findings of Hypothesis 1 indicates that perceived threats have a negative impact on public attitudes. When individuals believe that the recruitment of naturalized athletes poses a threat to their community, even if these threats are not personal, they tend to adopt more negative attitudes and distance themselves from these athletes as a means of alleviating the perceived threat. This finding aligns with existing research in immigration studies (Louis et al., 2013; Stephan et al., 2000; Ward and Masgoret, 2006), confirming that perceived threats are relevant when examining naturalized athletes as a distinct category of immigrants.

Conversely, the support of Hypothesis 2 demonstrates that perceived benefits are positively associated with favorable attitudes. Respondents who perceive greater benefits from the naturalization of athletes are more likely to be inclusive and supportive of these individuals, this might be because these people have always expected China’s success in elite sports especially of mega sport events like Olympics. Unlike typical international immigrants, naturalized athletes are often elite professionals chosen to represent their adopted countries in sports where those nations are traditionally less competitive on the global stage. As a result, citizens of these countries are more directly exposed to both the benefits and threats associated with the presence of these athletes. Therefore, strategies to improve public attitudes can be developed by effectively communicating the benefits of athletic naturalization. This approach not only enhances public acceptance but also contributes valuable insights to the broader field of immigration studies.

The support for Hypothesis 3 confirms a positive correlation between ABI and public attitudes, highlighting ABI as a significant factor influencing respondents’ attitudes toward naturalized athletes. It is intuitive that a positive perception of a naturalized athlete’s brand image would lead to greater likability and support, similar to the way people respond to other athletes. However, a more noteworthy finding is that ABI is also positively associated with respondents’ deep psychological acceptance of these athletes as members of the Chinese nation. This is particularly important in the context of China, which is known for its strong ethnic nationalism (Pu and Giardina, 2016). Existing studies suggest that an athlete’s recognition as a *bona fide* member of a nation typically depends on factors such as ancestral ties to the adopted country, being born or raised there, or fluency in the national language (Choi, 2020). However, the current study demonstrates that the public’s flexibility in embracing naturalized athletes can be significantly enhanced through a positive perception of their brand image. Embracing these athletes with a strong ABI could potentially elevate the status of the entire in-group.

The strong correlation between ABI and attitudes observed in this study may also be influenced by the context of the Winter Olympics. This event marked China’s first time hosting the Winter Games, and prior to this, China had not achieved the same level of success in winter sports as it had in summer sports. The success of naturalized athletes, such as Eileen Gu, who won two gold and one silver medal, contributed to China achieving its objective of participating in all sports and delivering its best performance ever at the Beijing Winter Olympics, as highlighted in *Outline of Building a Powerful Sporting Country* (GOSC, 2019). It is plausible that this significant success enhanced the correlation between the public’s perception of these naturalized athletes’ brand image and attitudes toward them. However, it remains to be seen whether this strong correlation would hold in sports where China traditionally excels (e.g., table tennis, gymnastics, or diving) or in sports that are closely tied to nationalism (e.g., men’s football, women’s volleyball). Further research is needed to confirm whether the effects observed in this study are consistent across different sports and contexts.

The findings related to Hypothesis 5 indicate that perceived benefits significantly influence people’s perceptions of the ABI of naturalized athletes. It is important to note that ABI is not an entirely objective measure; rather, it is shaped by respondents’ subjective awareness and perceptions of the athlete. Thus, beyond the athlete’s actual brand attributes, there are some other factors that can enhance how people perceive the athlete’s brand image. In this study, respondents who saw more benefits from athletic naturalization tended to have a more favorable perception of these athletes’ overall brand image.

In contrast, the rejection of Hypothesis 4 suggests that perceived threats do not necessarily lead to a diminished perception of naturalized athletes’ brand image. Even when respondents believe that athletic naturalization poses threats to the in-group, they might still evaluate these athletes similarly to other athletes, without derogating their ABI simply because of their special identity. This finding implies that naturalized athletes may be perceived differently from general international immigrants, as perceived threats often lead to prejudice against out-group immigrants.

Furthermore, this study proposed ABI as a mediator, and Hypothesis 7 confirms this mediating role between perceived benefits and attitudes. Perceived benefits not only directly influence attitudes but also enhance perceptions of ABI, which in turn leads to more favorable attitudes. This demonstrates a mechanism by which people develop their attitudes toward specific naturalized athletes.

Lastly, the support of Hypothesis 10 validates the moderating effect of Sport Event Involvement. SEI strengthens the relationship between ABI and attitudes, with the positive correlation between these two being notably stronger among respondents who were more involved with the Beijing Winter Olympics. This could be because those who were highly engaged with the event had greater exposure to naturalized athletes through various channels such as games, news reports, and advertisements. This increased exposure likely helped to solidify their perception of the athletes’ ABI, thereby leading to more favorable attitudes.

### 5.2 Managerial implications

China has already achieved significant success in elite sports through the naturalization of athletes, which has played a crucial role in its progress toward becoming a powerful sporting country. With the recruitment of more foreign-born sporting talents following the Beijing Winter Olympics, it is likely that athletic naturalization will become a long-term strategy. The findings of this study offer several implications for the governance of athletic naturalization practices.

First, this study confirms the relevance of perceived threats and benefits in shaping public attitudes toward naturalized athletes, who are viewed as a distinct category of international immigrants. To mitigate perceived threats, the entire process of athletic naturalization should be made more transparent. For example, providing public access to information about the criteria for naturalization, the assessment process, selection decisions, and associated costs could help reduce public concerns. Transparency in these areas would enable the public to better understand the rationale behind naturalization decisions, thereby alleviating fears and suspicions.

In addition to transparency, enhancing the perceived benefits of athletic naturalization is essential for fostering favorable public attitudes. It is crucial to assess and effectively communicate the true contributions of naturalized athletes to national teams and specific sports. This communication can be achieved through various channels, such as news reports, documentaries, and films, which highlight the successes and positive impacts of these athletes. By helping the public recognize the tangible benefits of naturalization, such efforts can contribute to a more inclusive and supportive environment for naturalized athletes. To this end, further research is needed to explore the long-term and sustainable success of athletic naturalization. For instance, studies could investigate how to maintain consistent success with naturalized athletes and how to leverage their popularity to draw public attention to both popular sports and less mainstream sports in China. Engaging more youth in these sports could help build a stronger foundation for future elite sports achievements and medal prospects. By focusing on transparency and effectively communicating the benefits of athletic naturalization, China can continue to strengthen its sporting prowess and foster a more positive public perception of naturalized athletes as valuable contributors to the national sports community.

Second, considering that ABI has the potential to significantly influence public attitudes and serves as a mediating factor between perceived benefits and attitudes, it is essential that the overall brand image of candidates be comprehensively assessed before their recruitment. Evaluating the full spectrum of a candidate’s brand image, rather than focusing on a single brand association, is crucial because candidates with a poor image are likely to garner less likability, identification, and support from the public. From the athletes’ perspective, careful management of their overall brand image is advisable to mitigate any potential opposition from the public. Since public perception of ABI is partly shaped by pre-existing knowledge and new information about specific naturalized athletes, it is also important to facilitate the construction of a more favorable ABI in the public’s mind by highlighting positive aspects of the athletes’ image through media channels.

Third, with the confirmation of the moderating effect of SEI on the relationship between ABI and attitudes, it becomes plausible to enhance the public’s SEI to foster a more comprehensive outlook on naturalization, thereby reducing exclusionary attitudes toward these athletes. To achieve this, efforts should be directed toward increasing public participation in sports and involvement in various sporting events, such as through China’s Fitness-for-All programs, which are likely to lead to more frequent and positive interactions with naturalized athletes. Additionally, leveraging the celebrity status of prominent naturalized athletes can further encourage public engagement with specific sports and events, ultimately fostering a more supportive environment for athletic naturalization.

### 5.3 Limitations and future research

This study has four major limitations that provide a foundation for future research on athletic naturalization. First, although respondents were free to select any athlete from the list provided, the majority chose Eileen Gu for assessment, which is unsurprising given her status as an Olympic gold medalist and prominent brand endorser. This concentration on a single athlete may limit the generalizability of the findings. To enhance the robustness and applicability of future studies, it would be beneficial to include a more diverse pool of naturalized athletes, enabling comparative and more extensive research.

Second, the data for this study were collected during the Beijing Winter Olympics, and given the cross-sectional nature of the study, it is limited by its inability to track changes in respondents’ perceptions of ABI and attitudes over time, particularly as enthusiasm for the event wanes. To address this limitation, future research could adopt a longitudinal approach, allowing for the monitoring of shifts in public perceptions over an extended period.

Third, while the mediating effect of ABI was confirmed, there may be other mediators at play. For instance, since the media is a primary source of information and contact between the public and naturalized athletes, the way media portray these athletes, given their special identities, might mediate public awareness of naturalization and attitudes toward these athletes. Future research should explore more variables as potential mediators in this context.

Fourth, sport event involvement in this study pertains to situational involvement. To gain a more nuanced understanding of the effects of involvement, future research should consider enduring involvement, such as general sport involvement or involvement with a specific sport. These constructs could provide deeper insights into how different types of involvement influence public attitudes toward naturalized athletes.

As the recruitment of foreign-born sporting talents is likely to continue, future research should explore athletic naturalization from various perspectives. Potential areas of study include the development of elite sports systems with naturalization, the impact of naturalized athletes on sports and business, the long-term social integration of naturalized athletes, and the dynamics of interaction between naturalized athletes and the general public. These directions will be crucial for understanding the broader implications of athletic naturalization in the context of both sports and society.

## Data Availability

The raw data supporting the conclusions of this article will be made available by the authors, without undue reservation.

## Footnotes

^1^ wenjuan.com
